# Effect of collagen sponge and fibrin glue on bone repair

**DOI:** 10.1590/1678-775720150374

**Published:** 2015

**Authors:** Thiago de Santana SANTOS, Rodrigo Paolo Flores ABUNA, Adriana Luisa Gonçalves de ALMEIDA, Marcio Mateus BELOTI, Adalberto Luiz ROSA

**Affiliations:** Universidade de São Paulo, Faculdade de Odontologia de Ribeirão Preto, Laboratório de Cultura de Células, Ribeirão Preto, SP, Brazil.

**Keywords:** Bone, Collagen sponge, Fibrin glue, Hemostatic agent

## Abstract

The ability of hemostatic agents to promote bone repair has been investigated using *in vitro* and *in vivo* models but, up to now, the results are inconclusive.

**Objective:**

In this context, the aim of this study was to compare the potential of bone repair of collagen sponge with fibrin glue in a rat calvarial defect model.

**Material and Methods:**

Defects of 5 mm in diameter were created in rat calvariae and treated with either collagen sponge or fibrin glue; untreated defects were used as control. At 4 and 8 weeks, histological analysis and micro-CT-based histomorphometry were carried out and data were compared by two-way ANOVA followed by Student-Newman-Keuls test when appropriated (p≤0.05).

**Results:**

Three-dimensional reconstructions showed increased bone formation in defects treated with either collagen sponge or fibrin glue compared with untreated defects, which was confirmed by the histological analysis. Morphometric parameters indicated the progression of bone formation from 4 to 8 weeks. Additionally, fibrin glue displayed slightly higher bone formation rate when compared with collagen sponge.

**Conclusion:**

Our results have shown the benefits of using collagen sponge and fibrin glue to promote new bone formation in rat calvarial bone defects, the latter being discreetly more advantageous.

## INTRODUCTION

Bone, in contrast with other tissues, can repair itself without scar formation. However, pathological fractures, great bone defects, insufficient blood supply, bone or surrounding tissues infections, and systemic diseases may negatively influence bone healing, resulting in delayed unions or nonunions^7^ ^,^ ^26^. The common therapeutic approach to treat these clinical situations is the use of bone autografts^5^ ^,^ ^8^.

Autograft bone is considered the “gold standard” due to the osteoconduction, osteoinduction and osteogenesis promotion as well as angiogenesis without the risk of disease transmission^21^. In oral and maxillofacial surgery, the areas from where autogenous bone can be harvested include extraoral sites as iliac crest, cranial vault and tibia plateau, and intraoral sites as mandibular symphysis, maxillary tuberosity, ramus, tori and exostoses^17^ ^,^ ^20^. However, the use of bone from these areas has some disadvantages, such as limited availability and increased morbidity associated with a second surgical procedure^6^.

Hemostatic agents have been used to control hemorrhage during surgical procedures for harvesting bone grafts from donor sites. Collagen sponge and fibrin glue are employed as hemostatic agents and as scaffolds for bone and cartilage tissue engineering^2^ ^,^ ^16^ ^,^ ^19^ ^,^ ^23^ ^,^ ^29^. Additionally, collagen sponge acts as wound burn dressing material and as intravaginal contraceptive diaphragm^2^ ^,^ ^14^. Applications of fibrin glue also include skin closure, vascular repair, bone piece fixation in fracture surgeries and fixation of biomaterials^11^ ^-^ ^13^ ^,^ ^22^ ^,^ ^25^.

In addition to hemostatic properties, it would be interesting if these materials could favor bone repair^4^. Indeed, the addition of a fibrin network to collagen sponge increased the osteoblast differentiation in a dose-dependent way, suggesting that these materials may favor bone repair^15^. Also, several studies have evaluated the influence of hemostatic agents on bone repair, with positive and negative results for collagen sponge and fibrin glue^4^ ^,^ ^13^ ^,^ ^24^. Thus, based on previous promising results, this study was designed to compare collagen sponge and fibrin glue in terms of bone repair in a rat calvarial defect model.

## MATERIAL AND METHODS

### Collagen sponge and fibrin glue

Collagen sponges (Gelfoam, Pfizer, São Paulo, SP, Brazil), derived from purified skin pig, were cut to produce samples with 5 mm in diameter and 2 mm height. This preparation was carried out in a laminar flow cabinet to keep the material sterile. Fibrin glue (Tissucol, Baxter, São Paulo, SP, Brazil), a combination of human fibrinogen, aprotinin and thrombin, was prepared immediately before using according to the manufacturer’s instructions. Solutions of fibrinogen and aprotinin were heated for 10 min at 37°C, mixed and kept for 1 min at 37°C. In another flask, a solution of thrombin was combined with calcium chloride and kept at 37°C. Mixtures of aprotinin with fibrinogen and thrombin with calcium chloride were placed in separate syringes connected by a Y-piece that allowed them to blend immediately before implantation in bone defect.

### Implantation procedure

All animal procedures were conducted under approval of the Committee of Ethics in Animal Research of the University of São Paulo. Thirty male Wistar rats weighting 250-300 g were anesthetized by a combination of ketamine (7 mg/100 g body weight) (Agener União, Embu-Guaçu, SP, Brazil) and xylazine (0.6 mg/100 g body weight) (Calier, Juatuba, MG, Brazil). The cranium of each rat was shaved and disinfected with povidone-iodine alcoholic solution (Rioquímica, São José do Rio Preto, SP, Brazil) and an incision was made along the sagittal suture to expose the parietal bones. Unilateral calvarial bone defects with 5 mm in diameter were created with a trephine under saline irrigation to remove both external and internal cortical and to preserving the dura mater. The bone defects were randomly treated (ten for each material) with either collagen sponge or fibrin, or kept empty (control). The skin was sutured with nylon 3.0 (Ethicon Ltd., São Paulo, SP, Brazil). After surgery, the animals received a single dose of antibiotics solution containing benzathine benzylpenicillin (156.000 IU/100 g body weight), benzylpenicillin procaine (78.000 IU/100 g body weight), benzylpenicillin potassium (78.000 UI/ 100 g body weight), dihydrostreptomycin base (65 mg/ 100 g body weight), and streptomycin base (65 mg/100 g body weight) (Fort Dodge, Campinas, SP, Brazil) and flunixin meglumine analgesic (10 mg/100 g body weight) (Schering-Plough, Vila Olímpia, SP, Brazil). At the end of 4 and 8 weeks, key time points to determine the progression of bone formation in this animal model, the rats were euthanized and the calvariae harvested and processed for histological and histomorphometric analyses.

### Histological analysis

Harvested calvariae were fixed in 10% buffered formalin for 36 h and decalcified in buffered EDTA (Merck, Darmstadt, Germany) for 10 days. Decalcified tissues were then embedded in paraffin and tissue sections with 6 µm thickness - from the central portion of samples - were stained with hematoxylin and eosin. Histological images were acquired under light microscopy (Axioskop 40, Carl Zeiss, Oberkochen, Germany) using a digital camera Axiocam ICc3 (Carl Zeiss) attached to the microscope and processed with the software Axion Vision (Carl Zeiss).

### Histomorphometric analysis

Before the decalcification for histological processing, samples fixed in 10% buffered formalin for 36 h, were submitted to micro-computed tomography (micro-CT) for morphometric analysis using the SkyScan 1172 system (Bruker-SkyScan, Kontich, Belgium). Images were acquired at 60 kVp and 200 mA and reconstructed using the software NRecon (Bruker-Skyscan) with smoothing 1, ring artifact correction 5 and beam hardening correction 20%. The micro-CT analyses were carried out using the 3D Ctan software (BruKer-Skyscan) to evaluate bone volume, percentage of bone volume, bone surface and trabecular number, thickness and separation.

### Statistical analysis

The results were expressed as mean±standard deviation (n=5) and compared by two-way ANOVA (treatment x time point) followed by Student-Newman-Keuls test when appropriated. The level of significance was set at p≤0.05.

## RESULTS

**Figure 1:**
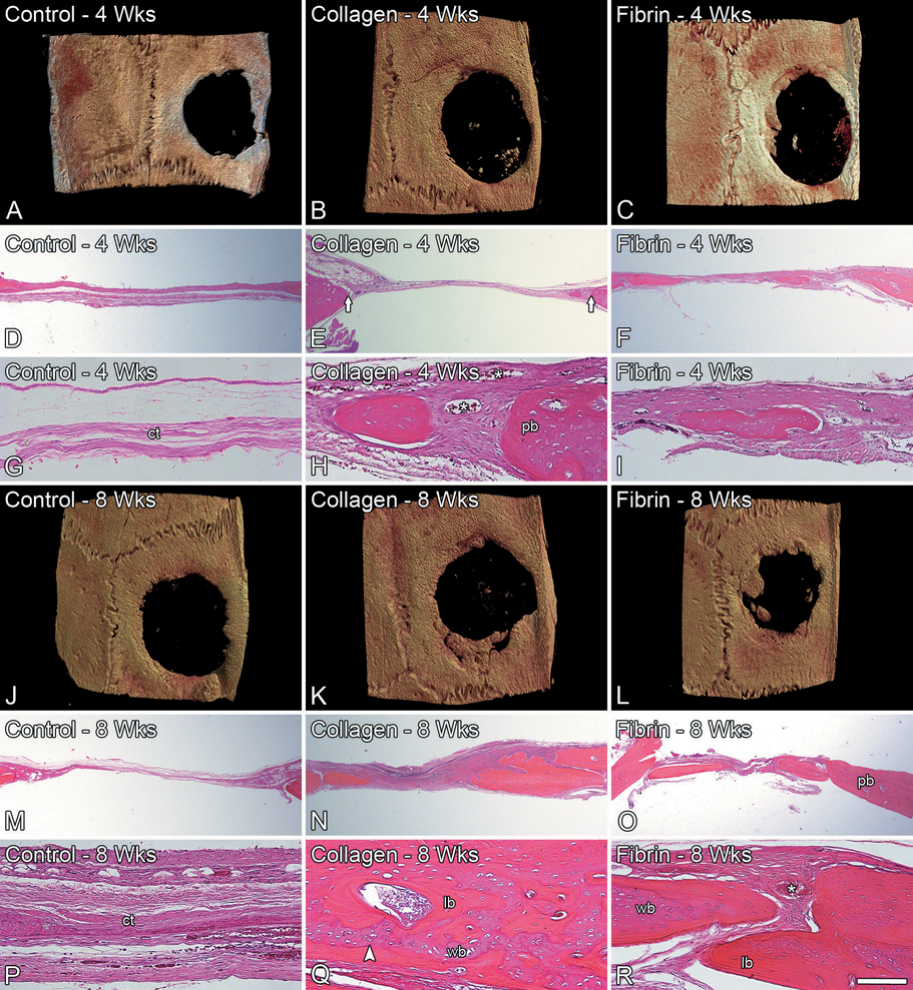
Three-dimensional reconstructed micro-CT images (A-C and J-L) and light microscopy (D-I and M-R) of untreated rat calvarial bone defects (control) (A, D, G, J, M and P) and the ones treated with either collagen sponge (B, E, H, K, N and Q) or fibrin glue (C, F, I, L, O and R), at 4 and 8 weeks. Arrows indicate the edge of the bone defect (E), asterisks indicate blood vessels (G-I) and arrowhead indicates cement line (Q). Hematoxylin-eosin stain. Scale bar: A-C and J-L=2.5 mm; D-F and M-O=800 µm; G,I=200 µm, H and P-R=100 µm. lb: lamellar bone, wb: woven bone, pb: parent bone, ct: conective tissue

Three-dimensional micro-CT reconstructions showed the lack of bone formation inside control defects at 4 and 8 weeks (Figure 1A and J) while some bone formation was observed in defects treated with either collagen sponge (Figure 1B and K) or fibrin glue (Figure 1C and L) at the same periods. In histological sections, we observed a connective tissue with no signs of bone formation inside control defects at both time points (Figure 1D, G, M and P). A histologically similar new bone tissue with homogeneously distributed osteocytes and partially lined by active osteoblasts was observed in defects treated with either collagen sponge (Figure 1E, H, N and Q) or fibrin glue (Figure 1F, I, O and R) at 4 and 8 weeks. Also, a cement line in the interface between this new woven bone and the lamellar one (Figure 1Q and R) and newly formed blood vessels were noticed in defects treated with collagen sponge or fibrin glue (Figure 1H and R). In addition, none of the defects exhibited foreign body reaction, chronic inflammation or infection.

**Figure 2:**
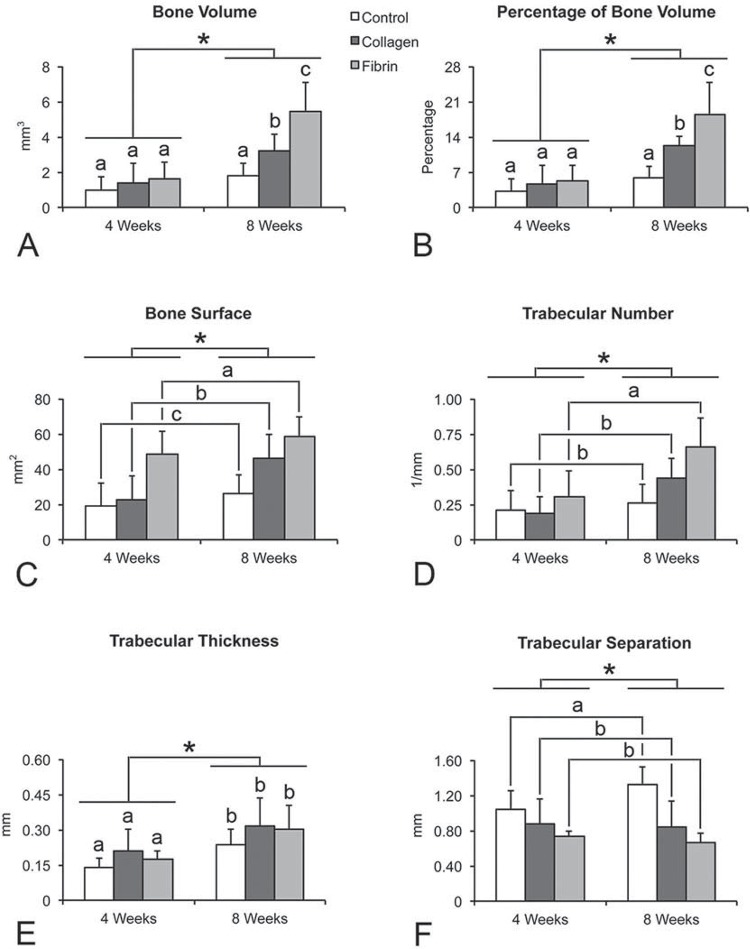
Morphometric parameters, bone volume (A), percentage of bone volume (B), bone surface (C), trabecular number (D), trabecular thickness (E) and trabecular separation (F), obtained from three-dimensional reconstructed micro-CT images of untreated rat calvarial bone defects (control) and treated with collagen sponge and fibrin glue, at 4 and 8 weeks. Data are presented as mean±standard deviation (n=5). Distinct letters indicate statistically significant differences among treatments and asterisks between time points

Bone volume (Figure 2A) and its percentage (Figure 2B) were affected by treatment (p=0.0001), time point (p=0.0001) and interaction treatment x time point (p=0.013). At 4 weeks, bone volume and its percentage had no statistically significant difference among treatments (p>0.05). At 8 weeks, bone volume and its percentage were higher (p<0.05) in defects treated with fibrin glue compared with collagen sponge, which were higher (p<0.05) than control defects. Additionally, bone volume and its percentage were higher (p<0.05) at 8 weeks compared with 4 weeks. Bone surface (Figure 2C) was affected by treatment (p=0.0001) and time point (p=0.006), but not by interaction treatment x time point (p=0.295). By taking 4 and 8 weeks together, bone surface was larger (p<0.05) in defects treated with fibrin glue compared with collagen sponge, which were larger (p<0.05) than control defects. Additionally, bone surface was larger (p<0.05) at 8 weeks compared with 4 weeks. Trabecular number (Figure 2D) was affected by treatment (p=0.005) and time point (p=0.0001), but not by interaction treatment x time point (p=0.116). By taking 4 and 8 weeks together, trabecular number was higher (p<0.05) in defects treated with fibrin glue compared with collagen sponge and control defects, which had no statistically significant difference (p>0.05). In addition, trabecular number was higher (p<0.05) at 8 weeks compared with 4 weeks. Trabecular thickness (Figure 2E) was affected by time point (p=0.0001), being larger at 8 weeks compared with 4 weeks, but it was not affected by treatment (p=0.120) and interaction treatment x time point (p=0.894). Trabecular separation (Figure 2F) was affected by treatment (p=0.0001), but not by time point (p=0.443) and interaction treatment x time point (p=0.118). By taking 4 and 8 weeks together, trabecular separation was larger (p<0.05) in control defects compared with defects treated with fibrin glue and collagen sponge, which had no statistically significant difference (p>0.05).

## DISCUSSION

This study was designed to evaluate if treatment with either collagen sponge or fibrin glue is able to favor bone formation in rat calvarial defects compared with non-treated defects. The newly formed bone observed was histologically similar irrespective of treatment, in contrast with the lack of detectable bone repair inside control defects. In general, morphometric parameters indicate more bone formation in treated defects when compared with the non-treated ones, with a tendency to be larger in defects treated with fibrin glue.

Histological sections are useful to observe and describe morphological features of tissues; however, they do not allow the reconstruction of three-dimensional images from serial sections, as a tissue segment is removed during the sample preparation^28^. Thus, in this study, the morphological description was based on decalcified histological sections and the morphometric analysis was performed on three-dimensional images obtained from micro-CT following established parameters.

In addition to the hemostatic effect, collagen sponge and fibrin glue may affect tissue healing including bone tissue. The use of collagen sponge on perforated cortical model promotes bone augmentation possibly by acting as a scaffold for cells and keeping the space for bone growth^27^. Also, collagen sponge has been successfully used as a BMP-2 carrier to enhance bone formation^3^ ^,^ ^9^ ^,^ ^10^. Despite the association with BMP-2 resulted in more bone formation, the effect of collagen sponge by itself could not be neglected^3^. Agreeing with these observations, our results clearly indicate that treatment with collagen sponge elicited better bone response compared with untreated defects. Regarding fibrin glue, the results are controversial since some studies have reported that fibrin glue either alone or associated with biomaterials may promote bone formation, whereas others did not show improvement in bone repair^1^ ^,^ ^13^ ^,^ ^18^. Our findings indicated that, as well as collagen sponge, fibrin glue favors bone formation compared with untreated defects. Despite the previous studies showing the positive effect of both collagen sponge and fibrin glue on bone repair, the comparison between them regarding potential of bone formation has been poorly explored. In this context, our results showed that fibrin glue promotes slightly more bone formation compared with collagen sponge. In keeping with our study, fibrin glue exhibits better osteogenic ability than collagen sponge as a BMP-2 delivery vehicle^30^.

In conclusion, we have shown, by histological and morphometric analyses, the benefits of using collagen sponge and fibrin glue to promote new bone formation in rat calvarial bone defects, with slight advantage to fibrin glue. Thus, these materials could be combined with cells and growth factors as either cell therapy or tissue engineering approaches aiming at enhancing and/or accelerating bone repair.
